# Current Therapeutic Approach to Acute Myocardial Infarction in Patients with Congenital Hemophilia

**DOI:** 10.3390/life11101072

**Published:** 2021-10-11

**Authors:** Minerva Codruta Badescu, Manuela Ciocoiu, Elena Rezus, Oana Viola Badulescu, Daniela Maria Tanase, Anca Ouatu, Nicoleta Dima, Ana Roxana Ganceanu-Rusu, Diana Popescu, Petronela Nicoleta Seritean Isac, Tudor-Marcel Genes, Ciprian Rezus

**Affiliations:** 1 Department of Internal Medicine, “Grigore T. Popa” University of Medicine and Pharmacy, 16 University Street, 700115 Iasi, Romania; minerva.badescu@umfiasi.ro (M.C.B.); daniela.tanase@umfiasi.ro (D.M.T.); anca.ouatu@umfiasi.ro (A.O.); nicoleta.dima@umfiasi.ro (N.D.); ana.ganceanu-rusu@umfiasi.ro (A.R.G.-R.); diana_ac_popescu@d.umfiasi.ro (D.P.); petronela-nicoleta_c_isac@d.umfiasi.ro (P.N.S.I.); ciprian.rezus@umfiasi.ro (C.R.); 2 III Internal Medicine Clinic, “St. Spiridon” County Emergency Clinical Hospital, 1 Independence Boulevard, 700111 Iasi, Romania; 3 Department of Pathophysiology, “Grigore T. Popa” University of Medicine and Pharmacy, 16 University Street, 700115 Iasi, Romania; manuela.ciocoiu@umfiasi.ro; 4 Department of Rheumatology and Physiotherapy, “Grigore T. Popa” University of Medicine and Pharmacy, 16 University Street, 700115 Iasi, Romania; 5 I Rheumatology Clinic, Clinical Rehabilitation Hospital, 14 Pantelimon Halipa Street, 700661 Iasi, Romania; 6 Hematology Clinic, “St. Spiridon” County Emergency Clinical Hospital, 1 Independence Boulevard, 700111 Iasi, Romania; 7 Department of Neurology, “Grigore T. Popa” University of Medicine and Pharmacy, 16 University Street, 700115 Iasi, Romania; tudor-marcel_m_genes@d.umfiasi.ro; 8 Neurological Rehabilitation Clinic, Clinical Rehabilitation Hospital, 14 Pantelimon Halipa Street, 700661 Iasi, Romania

**Keywords:** acute myocardial infarction, hemophilia, percutaneous coronary intervention, coronary stent, anticoagulant, antiplatelet therapy, antithrombotic therapy

## Abstract

Advances in the treatment of hemophilia have made the life expectancy of hemophiliacs similar to that of the general population. Physicians have begun to face age-related diseases not previously encountered in individuals with hemophilia. Treatment of acute myocardial infarction (AMI) is particularly challenging because the therapeutic strategies influence both the patient’s thrombotic and hemorrhagic risk. As progress has been made in the treatment of AMI over the last decade, we performed an in-depth analysis of the available literature, highlighting the latest advances in the therapy of AMI in hemophiliacs. It is generally accepted that after the optimal substitution therapy has been provided, patients with hemophilia should be treated in the same way as those in the general population. New-generation stents that allow short dual antiplatelet therapy and potent P2Y_12_ receptor inhibitors have begun to be successfully used. At a time when specific recommendations and relevant data are scarce, our study provides up-to-date information to physicians involved in the treatment of AMI in hemophiliacs.

## 1. Introduction

Major advances in the treatment of hemophilia have resulted in an increase in patients’ quality of life and life expectancy. Recent data have shown that from less than 30 years the median life expectancy increased to 59–71 years, 67–75 years, and 73–75 years in patients with severe, moderate, and mild hemophilia, respectively [1,2,3,4]. As cardiovascular risk factors and the atherosclerotic burden accumulate with age, hemophilia patients may develop acute vascular events as acute coronary syndromes (ACSs) and ischemic stroke.

Hemophilia has long been thought to have protective effects on the occurrence of acute ischemic vascular events because the hypocoagulant status of these patients prevents thrombogenesis [5]. Indeed, the mortality due to coronary artery disease (CAD) is lower in hemophilia patients compared to the general male population [2,6]. One study reported that even carriers of hemophilia are protected against fatal ischemic heart events [7]. Although major coronary events have been reported to be rare when they do occur, they result in high mortality. Soucie et al. showed that there was threefold higher mortality rate from acute myocardial infarction (AMI) in hemophilia patients compared to the general male population [5].

Acute vascular events like AMI and ischemic stroke are associated with hemophilia treatment also. Replacement of the deficient factor with large amounts of specific factor concentrates to achieve high levels as those required for major surgery or coronary invasive procedures (80–100%) have the highest thrombotic risk [8]. From 36 cases of AMI in hemophilia A (factor VIII deficient) patients, 22 cases were linked to treatment since they occurred during or shortly after the prothrombin complex concentrates, factor VIII concentrates, or recombinant factor VII (rFVII) infusion [9]. Although acute coronary events were scarce in the whole hemophilia A population, they had significant consequences. The mortality rate was high (seven fatal myocardial infarctions) and long-term consequences were major (seven cases of heart failure, one of which required heart transplant) [9]. Similar results came from an analysis of 13 cases of AMI in hemophilia B (factor IX deficient) patients. In eight cases there was a direct connection with hemophilia treatment: prothrombin complex concentrates infusion in five cases; and plasma, factor VIII inhibitor bypass activity (FEIBA), or cryoprecipitate infusion in three cases. Fatal AMI was recorded in three cases [10]. A large meta-analysis on thromboembolic events in hemophilia patients with inhibitors receiving activated prothrombin complex concentrate found 11 cases of AMI in 40 years of published data [11]. Recently, a new drug became available for the treatment of patients with hemophilia A with or without inhibitors. Emicizumab is a novel bispecific monoclonal antibody that bridges activated factor IX (FIX) with factor X (FX), activating FX, and thereby replacing the function of missing activated factor VIII (FVIII). Although emicizumab has shown marked efficacy and relative safety in clinical trials [12], one case of ST-elevation myocardial infarction (STEMI) was reported in relation to its use [13]. The acute coronary event occurred during emicizumab treatment, but also in the presence of multiple risk factors for thrombosis, including concomitant use of rFVII.

The first case of AMI in a hemophilia patient was reported in 1959 by Borchgrevink et al. [14]. Since then, many cases of AMI, independent or related to the hemophilia treatment, have been documented [9,15,16]. Although AMI is rare in hemophilia patients, it raises special therapeutic challenges. At present, percutaneous coronary intervention (PCI) and many types of coronary stents, anticoagulants, and antiplatelet agents are widely available, therefore, the management of a high bleeding risk patient requires a high level of expertise. The delicate balance between bleeding and thrombosis must be kept not only during the short and strictly monitored period of in-hospital stay, which is mandatory due to the acute coronary event, but especially early after discharge and in the long-term.

## 2. Cardiovascular Risk Factors and Atherosclerosis in Patients with Hemophilia

Before coagulation factor concentrates became available in the 1970s, the life expectancy of patients with hemophilia was no more than 30 years, with death caused by uncontrollable bleeding [17]. Therefore, atherosclerosis and its consequences had no clinical expression in those patients. Currently, due to the possibility of replacing the deficient coagulation factor (VIII or IX), life expectancy has increased significantly, approaching that of the general male population [1,2,17]. Age-related acute and chronic diseases not previously seen in this population emerged. Increasing prevalence of cardiovascular risk factors along with stable coronary artery disease, acute coronary syndromes, atrial fibrillation, and ischemic stroke are reported in adult and senior hemophilia patients.

Hemophilia, especially moderate and severe, has been thought to provide protection against the development of atherosclerosis. As coagulation cascade and inflammation are interconnected [18,19] and inflammation and thrombosis are contributors to the formation and progression of atheroma plaque [20,21], it has been hypothesized that in the case of factor VIII or IX deficiency, the patients are protected from developing atherosclerosis. Moreover, elevated FVIII levels were associated with the presence of atheroma plaques [22] and increased risk for ischemic heart disease [23,24,25] and stroke [26], thus low levels should provide some protection. There are few studies to support this hypothesis [27,28,29]. A study that included 50 men with moderate and severe hemophilia A showed that the prevalence of asymptomatic atherosclerotic plaques, evaluated by vascular ultrasonography, was significantly lower compared to the control group that had the same spectrum of cardiovascular risk factors—hypertension, hyperlipidemia, smoking habit and diabetes [29].

There is growing evidence that atherosclerosis is a process that affects blood vessels regardless of the presence or absence of hemophilia and the older the patients, the more advanced is the atherosclerosis. The contradictory results are mainly explained by the relatively young age of patients enrolled in previous studies (58.2 ± 10.3 [27], 48.3 ± 1.2 [28] and 41.72 ± 15.93 [29]) and to a lesser extent by the heterogeneous nature of the enrolled population. Some studies included not only hemophiliacs but also patients with hypocoagulability states due to deficiencies of other factors, as with the von Willebrand disease [27,28]. Recent studies, including only hemophilia patient and using ultrasound evaluation, along with high-performance imaging techniques, demonstrated that they can suffer from atherosclerosis to a similar extent as the general population.

Biere-Rafi et al. performed a study that evaluated asymptomatic atherosclerosis enrolled obese and non-obese hemophilia A patients and non-hemophilic controls, without cardiovascular disease [30]. The carotid and femoral intima-media thickness (IMT) were assessed by vascular ultrasonography. As expected, the presence of obesity increased the cardiovascular risk and the IMT was higher in obese subjects than in non-obese ones. However, in non-obese subjects, no difference in IMT was observed, supporting the hypothesis that hemophilia patients have the same risk for developing atherosclerotic plaques as the general male population.

The severity and the extent of coronary artery calcification (CAC) strongly correlate with the degree of atherosclerosis [31], the risk of further cardiovascular events, and cardiovascular death [32]. CAC assessed by low-dose computed tomography scan is the main noninvasive tool for the evaluation of the coronary arteries atherosclerotic burden. Using this imagistic technique, Tuinenburg et al. evaluated 42 patients with severe or moderate hemophilia A, aged 66.5 ± 4.6 years, and found no difference in the extent of coronary artery atherosclerosis compared to control [33]. However, 24% of the subjects in both groups had severe calcification of the coronary arteries, a rate that questions the protective role of factor VIII deficiency in the development of atherosclerotic plaques. Zwiers et al. performed a study focusing on the presence and extent of asymptomatic atherosclerosis assessed by both CAC score, and carotid ITM in hemophilia patients confirmed the results of previous studies. Moreover, it showed that there were no differences depending on the type of hemophilia [34].

A strong validation of the imaging examinations was offered by the results of 14 autopsies on hemophilia A patients. It was shown that intraluminal coronary stenosis did not differ in hemophiliacs compared to the non-hemophilic control group [35].

Thus, considering that cardiovascular mortality is lower in hemophiliacs compared to the general population, but the amount of coronary artery atherosclerosis is similar, a possible explanation was initially sought in the direction of the prevalence of cardiovascular risk factors. Nonetheless, both older [36] and newer studies [37,38,39,40,41,42] showed that the prevalence of cardiovascular risk factors is as least as high in hemophiliacs as in the general population, if not higher.

In a cohort of 709 hemophilia patients from North-European countries (the Netherlands and the United Kingdom), hypertension was more prevalent in hemophiliacs than in the general population, diabetes mellitus and smoking were similar, while obesity and hypercholesterolemia were less frequent [40]. One large European study, enrolling 532 hemophilia patients over 40 years of age from 13 countries, showed that among the cardiovascular risk factors, hypertension and smoking were significantly more prevalent (45% and 49.7%, respectively) than obesity and diabetes mellitus (13% and 10%, respectively) [43]. An Italian study that included only patients with severe hemophilia and over 60 years of age showed that these men had less hypercholesterolemia, hyperglycemia, and overweight, but similar rates of hypertension as age-matched individuals from the general population [41].

Although there are differences in the ethnic spectrum of the population and the prevalence of cardiovascular risk factors between continents, the leading position of hypertension in hemophiliacs is maintained in American studies also. In a large north-American cohort of 2506 males with hemophilia A, hypertension and dyslipidemia were found in 22.6% and 15.9% of cases, respectively. Both cardiovascular risk factors were more prevalent in hemophiliacs than in age-matched men from the general population, in which 15.5% and 11.9% of individuals had hypertension and dyslipidemia, respectively [38]. Moreover, cardiovascular comorbidities were more prevalent and appeared earlier in life in hemophiliacs than in the general male population [38]. Furthermore, a US study that enrolled 200 men with moderate and severe hemophilia, aged between 54 and 73 years old, showed a high prevalence of cardiovascular risk factors [42]. More than half of the hemophiliacs had hypertension (64.4%) and dyslipidemia (65.8), half were smokers (48.5%), nearly one-third were obese (29.5%), and one-fifth diabetics (19.5%). As by design, this study had age-comparable men to the ARIC (atherosclerosis risk in communities) study, and it showed a higher prevalence of hypertension in hemophiliacs than in the general male population. No difference in cardiovascular risk factors by the type of hemophilia was observed.

Among the modifiable cardiovascular risk factors, hypertension has been the world’s leading contributor to cardiovascular disease for 20 years [44]. Importantly, many studies have highlighted that hypertension is even more prevalent in hemophiliacs than in the general population, therefore, active screening for hypertension and early initiation of treatment should be a priority in the prevention of cardiovascular disease in hemophiliacs.

It is interesting that, although in a state of hypocoagulability, hemophilic patients may develop both arterial and venous thrombosis and most arterial thrombosis is coronary, leading to myocardial infarction or unstable angina. A large retrospective analysis found that in patients with hemophilia A, arterial thrombosis was 3.72 times more prevalent than venous thrombosis, while in hemophilia B it was just as common [45]. In the whole hemophilic population, the ratio between arterial and venous thrombotic events was 2.5, suggesting that the protective effect of coagulation factor deficit is more pronounced on venous thrombosis than on arterial thrombosis [46]. The full explanation of this phenomenon is still unclear.

Given that hemophiliacs do have less cardiovascular risk factors or atherosclerotic burden than the general male population, their lower cardiovascular mortality could be explained by the decreased risk of arterial thrombosis. That puts the atheroma plaque into a central position. One important issue to consider is that hemophiliacs have more stable atheroma plaques due to reduced thrombin generation [21,39,47]. Another hypothesis is that, due to hypocoagulability, it is less possible to develop occlusive vascular thrombosis when plaque atheroma ruptures or erodes [6,39], but not all arterial occlusions result from the presence of a thrombus. The slow growth of atheroma plaques to critical coronary stenosis through lipids accumulation and intraplaque hemorrhage should be considered. Other events such as plaque rupture with distal atheroembolism or coronary vasospasm may lead also to acute coronary events [35].

## 3. Acute Myocardial Infarction in Patients with Hemophilia

Coronary artery disease has an increasing prevalence in the hemophiliacs worldwide. When compared to age-matched patients from the general population, North-American individuals with hemophilia A have significantly more CAD [38]. A multicenter European study showed that 6.1% of hemophiliacs had CAD and that the disease was present regardless of the severity of hemophilia. Regarding the patients with mild, moderate, and severe hemophilia, 9%, 7%, and 4.6% had CAD, respectively [43], but when only individuals with moderate and severe hemophilia were considered, the prevalence of CAD was lower than in sex-matched patients from the general population [41,42], confirming that CAD is far more frequently encountered in individuals with mild disease.

The results of an international, retrospective, 10-year survey (2003–2013) enrolling 2380 adult hemophilic patients showed that ACS are very rare events in this population [48]. During the study period, only 20 events were identified. It was noted that 37% of patients with ACS were under 50 years of age and that more than half of the hemophiliacs (53%) had at least three cardiovascular risk factors at the time of the acute coronary event. It became very clear that early implementation and even intensification of the primary prevention measures are of major importance in these patients [48].

In 2018, the fourth universal definition of myocardial infarction was published [49]. Briefly, type 1 AMI is the consequence of acute thrombosis on a ruptured or eroded atherosclerotic plaque, while type 2 AMI occurs in the context of a mismatch between oxygen supply and demand. Acute bleeding with a precipitous drop in hemoglobin level may result in AMI in patients with stable known or presumed coronary artery disease through a supply-demand oxygen imbalance. Type 3 AMI includes sudden cardiac death presumed of ischemic origin and AMI detected by autopsy. Type 4 and 5 AMI are related to coronary procedures (stent or scaffold thrombosis, PCI or coronary artery bypass grafting) [49].

A systematic review that included all cases of AMI in patients with hemophilia A published between 1954 and 2005 identified 36 events [9]. In 22 cases the AMI was related to the substitution treatment with coagulation factors. When available, the autopsy reports commonly showed extensive atherosclerotic lesions and transmural hemorrhage [50]. Fresh thrombus formation was found only in a few cases [9]. However, as most cases of AMI occurred in conditions of elevated levels of coagulation factors and/or in the presence of activated factors in the infused preparation, we consider that they were favored by the intense prothrombotic environment. This is the case of 13 patients with AMI up to 40 years old, to which the acute coronary events were related to substitution treatment with coagulation factors or desmopressin administration. Similar data is provided by another systematic review that includes all cases of AMI in patients with hemophilia B published until 2005 [10]. Out of a total of 13 identified cases, 8 cases were related to the substitution treatment with coagulation factors. Only one patient was under 40 years of age.

Additional data is provided by recently published coronary angiography reports describing significant or critical stenosis of one or more vessels [51,52,53,54,55] or acute thrombotic occlusions [53,56,57]. Intrastent thrombosis as a cause for AMI was also reported [58].

Severe bleedings in hemophiliacs with comorbidities may lead to AMI through a supply–demand oxygen imbalance. A hemophilic patient with advanced liver disease, esophageal varices, and cardiovascular risk factors experienced severe gastrointestinal bleeding with hemorrhagic shock. His electrocardiogram confirmed the AMI, which occurred due to hypoperfusion [59].

Over the past decade, the treatment of AMI has constantly improved. We conducted this research to assess the extent to which progress has changed the approach to AMI in hemophiliacs. As data on the presentation and management of AMI are scarce and mainly come from isolated case reports and a short series of patients, it is difficult to get a precise overview. Our analysis focused on the current management of myocardial infarction in hemophilia patients considering the latest advances in antithrombotic, interventional, and surgical therapies. A search in Web of Science was performed using “(myocardial infarction or acute coronary syndrome) and (hemophilia or haemophilia)”. The keywords were searched in the titles and abstracts of the articles. The search was restricted to articles published from 2013 to date. This time frame was chosen considering that the purpose of our work was to make an in-depth analysis of current therapy and that Applicability of the European Society of Cardiology guidelines on the management of acute coronary syndromes to people with hemophilia—an assessment by the ADVANCE (age-related developments and comorbidities in hemophilia) Working Group was published in 2013 and has not been updated since [60]. Although institutional guidelines [61,62], a consensus review [8], and reviews that focus on current concepts [63,64] are also available, none of them have a guideline status. We manually screened the titles and abstracts of all 291 articles retrieved from the automatic search. Articles referring to acquired hemophilia were excluded because the effects of long-term coagulation factor deficiency are different from that of short-term ones. Articles in other languages than English were also excluded.

### 3.1. Selection of the Reperfusion Therapy

The expeditious opening of the culprit vessel has the highest priority in the treatment algorithm of AMI. Current guidelines recommend early mechanical revascularization with PCI as the first therapeutic option for AMI treatment in the general population [65,66]. Particular for the STEMI patients, systemic fibrinolysis should be the initial reperfusion strategy if the time to PCI is estimated to exceed 120 minutes. The fibrinolytic treatment should be administered within 10 min from the STEMI diagnosis [65]. The patient should then be referred to a PCI center for routine early angiography or rescue PCI in the case of failed fibrinolysis [65].

PCI and fibrinolysis are both procedures associated with significant bleeding risk, therefore, the AMI therapeutic approach is very challenging in hemophilic patients. Moreover, there are no evidence-based guidelines for their antithrombotic management, as congenital bleeding disorders represented exclusion criteria in ACS clinical trials. Only expert consensus [8,60] and institutional guidelines recommendations [61,62] are available.

PCI is the main line of treatment for AMI in hemophiliacs and it should be performed as soon as possible. Additional therapeutic measures such as supplementation of the deficient coagulation factor are necessary because the hemorrhagic risk will further increase during and after PCI due to antithrombotic therapy. Data extracted from a large North American database showed that hemophiliacs had similar outcomes as controls following PCI for ACS, regardless of the type of disease, suggesting that PCI is generally well tolerated. Moreover, the increased risk of bleeding did not contribute to mortality during hospitalization [67].

Fibrinolysis is generally contraindicated in patients with bleeding disorders, including hemophiliacs. Still, when primary PCI is not available in the timeframe recommended by guidelines, systemic fibrinolysis may be justifiable in hemophiliacs with STEMI, but only under adequate coagulation factor replacement therapy [60]. However, this approach has many disadvantages, and as far as we know, its use has not been reported in the last decade [48]. This can be explained by the wide availability of PCI at this time and by differences regarding the hemorrhagic events. One large meta-analysis focusing on bleeding events associated with primary PCI and fibrinolysis in STEMI patients from the general population concluded that although overall bleeding complications during a 30-day period were almost similar, there were significant differences in the localization of hemorrhagic events [68]. Fibrinolysis was associated with a significantly higher risk of intracranial bleeding than PCI. Other outcomes that favored PCI over fibrinolysis were the lower rates of death, reinfarction, and stroke.

Coronary artery bypass grafting (CABG) usually assumes a time delay. Because in AMI, “time is muscle” CABG is currently used in only 5% of AMI patients, it is reserved for patients with complex coronary artery disease as a three-vessel disease, left main stenosis or stenosis of the proximal left anterior descending artery [60]. Data from an international, retrospective, 10-year survey (2003–2013) enrolling more than 2000 hemophiliacs reported that all four cases of STEMI were treated with PCI while the eight non-ST segment elevation myocardial infarction (NSTEMI) cases were managed as follows: PCI in one case, CABG in two cases, and medical treatment in five cases [48]. Revascularization either by PCI or CABG was successful in all cases and without excessive bleeding during initial management, but none of the patients who underwent CABG had severe hemophilia. Indeed, due to the increased complexity and possible poor procedural outcomes, CABG is rarely a therapeutic option for patients with severe hemophilia [48,63]. Topaloglu et al. published the case of a patient with mild hemophilia A, who presented with STEMI and underwent CABG due to multi-vessel disease not amendable by PCI. The surgery was performed under the same CABG protocol used in the general population, along with prophylactic administration of FVIII 10 days before and 11 days after the procedure [69]. Successful elective CABG in hemophiliacs was also reported [70,71], and it was highlighted that intense bleeding prevention measures should be taken. Factor levels were normalized for 7–10 days before CABG, with trough levels of 70–80%. In patients with severe hemophilia, high-dose factor prophylaxis with trough levels of 20–30% was administered for 3 weeks after the procedure [71].

In the past, many patients were treated conservatively. Results of a retrospective analysis (1998–2011) of a large North American database showed that hemophiliacs are more often managed medically during acute hospitalization than the general population, suggesting avoidance of PCI and CABG in this population for fear of bleeding [67]. In the Danish cohort of hemophiliacs, four of five patients with AMI treated before 2013 were managed conservatively. In two cases, CABG was performed after 2 and 9 months after the acute event, respectively [72]. Peng et al. reported the successful management of a difficult case of NSTEMI complicated with respiratory and cardiac arrest in a patient with severe hemophilia A [73]. However, few cases of AMI are treated conservatively today because modern techniques are widely available.

### 3.2. Selection of the Anticoagulant

During PCI, the anticoagulant treatment is mandatory, even in hemophiliacs. Several anticoagulants can be used: unfractionated heparin (UFH), enoxaparin, and bivalirudin [65,66]. Due to the increased risk of bleeding in these patients, it is preferable to use anticoagulants with shorter than longer half-lives, reversible instead of irreversible, and in continuous infusion instead of bolus injections [64]. Although placebo-controlled trials evaluating UFH in primary PCI have not been conducted, UFH is recommended as the anticoagulant of choice during PCI [65] based on the large experience with this agent and on the following advantages: short half-life, rapid reversibility with protamine sulphate, and easy assessment of the anticoagulant effect. Before PCI, a single bolus infusion of UFH of 70–100 IU/kg is indicated, without additional UFH administration after the procedure [61].

In the catheterization laboratory, the level of anticoagulation is monitored using activated clotting time (ACT) levels. It is the most commonly used parameter to monitor UFH administration during PCI. Being a point-of-care test, the result at ACT is quickly available. ACT is prolonged in patients with hemophilia, but replacing the deficient coagulation factor suppresses this phenomenon. Thus, ACT levels remain reliable to monitor coagulation and to guide UFH dosage during PCI [55]. Target values are not different in hemophiliacs than in the general population.

Enoxaparin and bivalirudin are infrequently used. Although the low molecular weight heparins (LMWH) are associated with lower rates of bleeding complication and heparin-induced thrombocytopenia than UFH, their effect is harder to control and reverse, hence the preference for UFH as a safer choice [74]. The short-acting direct thrombin inhibitor bivalirudin came with several advantages as inhibition of both circulating and clot-bound thrombin, no need for monitoring, and a low rate of bleeding complications. However, its use is limited due to the evidence of an increased rate of acute intrastent thrombosis, in the first 24 h after PCI, compared to UFH [75,76,77]. Only a few case reports on bivalirudin use in hemophilic patients with AMI treated with PCI were published to date [8,74,78,79].

The use of anticoagulants in patients with hemophilia without replacement therapy is contraindicated, therefore, expeditious administration of coagulation factor concentrates is needed. The factor replacement therapy should be given in parallel with ACS-specific treatment, but it is needed that the replaced factor achieve a peak level of at least 80% prior to sheath removal [60]. If the administration of replacement therapy is performed before PCI, it is acceptable that the procedure is delayed only with the time required for the coagulation factor concentrate to be administered. Infusion is preferred over bolus because acute thrombosis may occur due to a rapid increase in factor levels [16,56]. It was considered that within 24 h after PCI, trough levels should be around 50% [60], but recent data suggest that replacement coagulation factor therapy should aim for a peak level of at least 80% for 48 h after PCI [8,17,62,63,80,81,82]. The amount of factor to be infused depends on the severity of the hemophilia. To determine the level of factor VIII or IX in the laboratory takes considerable time, and delaying the interventional treatment that must be performed in a timed manner can result in severe myocardial damage or even death. In this setting, the patient’s last known factor level can be used to guide the administration of the replacement therapy. Ideally, the patient’s baseline factor level should be easily available without the need for measurement in emergency conditions [60]. It must be acknowledged that the deficient coagulation factor level is primarily responsible for both the bleeding risk and the clinical severity of the bleeding [8].

The above recommendations were formulated based on the predictable response of patients with hemophilia without inhibitors to coagulation factor replacement therapy [60]. Patients with inhibitors require administration of bypassing agents and they experience a much less predictable hemostatic response to therapy [80], therefore, it is more difficult to reliably prevent or treat bleedings. The close collaboration with an experienced hematologist is mandatory, in order to appropriately guide the replacement therapy.

In the rare occasions when patients were managed conservatively, LMWH was used, with a preference for enoxaparin, but in the Danish study, dalteparin was the LMWH of choice, administered in two cases for 3 and 4 months after the acute coronary event, respectively [72].

### 3.3. Selection of the Access Site for PCI

Bleedings at the access site are a major concern since they represent 40% of all causes of bleeding in ACS patients undergoing emergency PCI [83]. Many are minor, as local hematomas, without clinical significance or requiring special intervention, but others can be major and lead to many disadvantages, such as hemodynamic instability, need for blood transfusion, and even antithrombotic drug discontinuation. 

The femoral artery has been the preferred vascular access for PCI for a long time. Despite the experience gained, this procedure is burdened by vascular complications such as bleeding at the site of arterial puncture and the occurrence of arterial pseudoaneurysms and arteriovenous fistulas. The severity of bleeding after transfemoral PCI varies from small and localized inguinal hematomas to major or life-threatening hemorrhages of the groin, retroperitoneal, and rectus abdominis [84]. While in the initial observational studies the rates of major or clinically significant bleeding exceeded 10%, currently the rate is halved due to the use of smaller sheaths, reduced intensity and duration of periprocedural anticoagulation, and shorter duration of the procedure [85]. Femoral access was successfully used for PCI in patients with hemophilia [86]. Out of 54 males undergoing coronary angiography with or without PCI, 52% had femoral catheterization [81]. Three patients (6%) recorded major bleeding events: two with bleeding at the femoral access site [48,87] and one with intramuscular hemorrhage [52]. Other studies have also reported negligible complications associated with transfemoral PCI in hemophiliacs [53,88]. This low rate of bleeding complications suggests that interventional cardiologists who treated these patients had extensive experience in performing femoral catheterization. Therefore, transfemoral PCI is a viable solution for the hemophiliacs if the interventional cardiology center has experience in using this access route.

Several methods are available to achieve hemostasis at the arterial puncture site: manual or mechanical external compression, internal vascular closure devices, and procoagulant drug pads applied to the skin before manual compression is performed [89]. Manual compression is the traditional technique for achieving hemostasis at the vascular puncture site. Despite its high rate of success and low rates of vascular complications, it requires close observation, a prolonged period of immobilization, significant personnel time, and causes discomfort for both the patient and practitioner [90]. The use of procoagulant drug pads has the advantage of shortening the manual compression time [89] and reducing the incidence of bleedings and vascular complications [91]. Studies with compression devices demonstrate successful use in nearly all patients with generally equal efficacy. Mixed results have been reported in terms of femoral vascular complication rates, especially the higher incidence of hematoma [90]. Vascular closure devices reduce the necessary time for hemostasis, the complication rate and the bed-rest time [92], and improve patient comfort in comparison with manual compression [93,94,95]. In 13 hemophiliacs undergoing transfemoral coronary angiography, successful hemostasis was achieved with manual compression (nine cases) and vascular closure devices (four cases) [81]. Other data also supports the use of vascular closure devices [96,97]. A preference for a suture-based closure device or collagen plug-based device is highlighted especially when large French sheaths are used for catheterization [98].

A decade has already elapsed since the publication of RIVAL (radial versus femoral access for coronary angiography and intervention in patients with acute coronary syndromes) and RIFLE-STEACS (radial versus femoral randomized investigation in ST-elevation acute coronary syndrome) trials [83,99]. Compared to femoral access, radial access was associated with lower incidence of bleeding at the arterial puncture site and vascular access complications (thrombosis, dissection, aneurysm), shorter intensive care unit and hospital stay, and lower rates of cardiac mortality at 30 days. These findings were further reinforced by the results of the MATRIX trial (radial versus femoral access in patients with acute coronary syndromes undergoing invasive management) [100]. The advantages of radial access compared to the femoral one were highlighted once again. The radial access was associated with fewer major bleedings and all-cause mortality, thus it reduced the net adverse clinical events [100]. An Italian study of unselected patients with AMI treated by PCI found that not only the bleeding rates and vascular complications were lower, but also mortality at 2 years decreased when using arterial instead of femoral access [101].

As the use of radial access in patients undergoing primary PCI for AMI is associated with better safety, lower morbidity, and cardiac mortality, it is currently recommended by the guidelines [65,66,102] as the default access site in the general population of AMI patients undergoing primary PCI. This strategy mainly aims to reduce the bleeding risk related to the invasive procedure, therefore, it is the best approach for PCI in hemophilia patients as well [60]. Although the radial access is recommended in patients with high bleeding risk, such as hemophiliacs, it should be performed only by interventional cardiologists with experience in this technique. Otherwise, the expected benefit of radial access will not be achieved [61].

Several aspects regarding the utility of radial access are to be considered in hemophiliacs. First, the radial artery is of smaller caliber and is more superficial than the femoral artery, so the compressive hemostasis measures are easier to do and more effective. A 60% reduction in puncture-related bleeding events was obtained using radial instead of femoral artery access [64]. Second, iatrogenic bleeding in non-compressible sites (e.g., retroperitoneal hemorrhage) must be avoided, as they could require aggressive therapeutic measures, including supplementation of the deficient coagulation factor. The administration of coagulation factors was itself involved in triggering acute coronary thrombotic events [16,56,103,104], therefore, the premises for their intensive use must not be created. 

Ensuring correct hemostasis at the puncture site after sheath removal is mandatory in order to reduce local complications, both bleeding (too little compression) and radial artery occlusion (too much compression). The use of a compression device after completing the procedure may facilitate the bleeding control as it exerts a more stable and continuous pressure on the artery, and is free from manual compression drawbacks: time and personnel consuming, inconstant pressure due to operator hand fatigue, and risk of occlusive radial artery compression [105]. Currently, there are many dedicated compression devices available on the market, most of them in the form of wristbands that exert a controlled and adjustable compression to the radial artery. If a device is used for hemophiliacs, the best is one that allows direct viewing of the puncture site through the transparent observation window.

While access-site bleeding complications and radial artery occlusion do not significantly depend on the technique used for hemostasis [106], there are currently no guideline recommendations on prioritizing one approach over the other, but, as the time required to obtain hemostasis varies significantly with the method, it must be known that time to achieve hemostasis is significantly shorter if the manual technique is used [106].

### 3.4. Selection of Stent

In patients presenting with AMI and undergoing PCI, a stent is usually implanted to treat the culprit lesion. For many years, bare-metal stents (BMS) were the only option available. Once drug-eluting stents (DES) were developed they became widely used because they outperformed BMS. DES are coated with anti-proliferative agents, hence they are associated with reduced risk of restenosis and stent thrombosis [107], thus the need for target-vessel revascularization is lower when using DES than BMS. Furthermore, DES are associated with lower rates of target-vessel reinfarction and cardiac death than BMS [108,109].

Since the re-endothelialization is delayed, the necessary time of dual antiplatelet therapy (DAPT) is longer [110]. Current international guidelines recommend DAPT after DES implantation in ACS patients for at least 12 months if the bleeding risk is low and for at least 6 months if the bleeding risk is high [111,112]. While BMS need only 4–6 weeks of DAPT, they remained indicated in selected high bleeding risk patients requiring time-limited DAPT [110].

For hemophilia patients undergoing primary PCI, the use of BMS over DES was recommended by the ADVANCE Working Group [60], considering that the endothelialization time, the interval with risk of intrastent thrombosis, and the duration of dual antiplatelet therapy are shorter, thus limiting the period with high bleeding risk. The use of DES in hemophilic patients was to be considered only in cases with symptomatic restenosis [53] or when a high risk of restenosis due to diabetes existed [8,60]. There was also the opinion that hemophiliacs with mild disease—factor levels exceeding 25%—could receive DES as they may not require administration of coagulation factor concentrates for the duration of DAPT [61].

The new generations of DES (everolimus-, zotarolimus- and biolimus A9-eluting) have features that may change these recommendations in future guidelines. Everolimus-eluting stents (EES) are associated with lower rates of stent thrombosis, reinfarction, and target-lesion revascularization compared to the first-generation DES [108,113]. In one comparative trial, zotarolimus-eluting stents (ZES) and EES showed similar efficacy and safety at 5-year follow-up [114]. Biolimus A9 (also known as umirolimus) coated stents (BES) are polymer-free DES associated with superior clinical outcomes compared with BMS and first-generation DES [115]. Moreover, BES have similar rates of reinfarction, target-vessel revascularization, and cardiac death as second-generation durable-polymer DES [115]. With so many advantages, it is standard practice to stent infarction-related artery with contemporary DES [65,66,116,117]. Experience in using these new stents in patients with ACS also included hemophiliacs. Carbone et al. successfully implanted two ZES in a patient with unstable angina and mild hemophilia A, with a good outcome at 6-month follow-up while on DAPT [118]. Kacprzak et al. implanted four EES and one BES in a difficult STEMI case. DAPT for 12 months and then SAPT were recommended, and a good outcome was reported at 30-month follow-up [119]. Bailly et al. used a ZES in a STEMI patient with severe hemophilia A and DAPT for more than 12 months [51].

Recently, published results of two randomized control trials showed that ZES and BES had superior efficacy and safety to BMS when used in patients at high risk of bleeding who were treated with DAPT for a short time after PCI [120,121]. ZES was superior to BMS in reducing stent thrombosis, reinfarction, and target-vessel revascularization in patients at high risk of bleeding receiving DAPT for 1 month [120]. Compared with BMS, BES significantly reduced the need for target-vessel revascularization and a composite of cardiac death, reinfarction, or stent thrombosis at 1-year follow-up in patients with high bleeding risk receiving DAPT for 1 month [121]. Recent data from a comparative study showed that ZES was non-inferior to BES in terms of safety and efficacy when used in patients at high risk of bleeding who received DAPT 1 month after PCI [122].

The use of third-generation DES with 1 month DAPT after PCI was recently reported in hemophilic patients as well [71]. Theodoropoulos et al. reported the successful use of ZES in a 70-year-old patient with NSTEMI, followed by DAPT with aspirin and clopidogrel for 1 month, without any complication [55]. Offering the efficacy of a DES with 1 month DAPT as BMS, contemporary DES are successfully used in hemophilic patients with ACS undergoing primary PCI (Table 1).

### 3.5. Selection of the Antiplatelet Therapy

There are several challenges in hemophilia patients undergoing primary PCI for AMI. The first to consider is the administration of loading doses of antiplatelet agents before PCI; the second is the DAPT duration after stent implantation; and the third is the choice of the P2Y_12_ receptor inhibitor.

In the general population of STEMI patients undergoing primary PCI, to administer aspirin and a P2Y_12_ receptor inhibitor in a loading dose prior to the procedure is the standard of care. The P2Y_12_ receptor inhibitor is given before or at the time of PCI at the latest. Prasugrel and ticagrelor are preferred over clopidogrel, as they have a more rapid onset of action and offer greater and more consistent inhibition of P2Y_12_ receptor than clopidogrel [123]. Moreover, in randomized clinical trials, they were superior to clopidogrel in clinical outcomes. Prasugrel showed lower rates of ischemic events, including stent thrombosis than clopidogrel [124]. Ticagrelor reduced the rate of myocardial infarction, stroke, or death from vascular causes better than clopidogrel [125]. However, all P2Y_12_ receptor inhibitors are associated with bleeding risk, higher with prasugrel and ticagrelor than with clopidogrel, therefore, they should be managed with caution. The use of prasugrel and ticagrelor should be avoided in patients with an unacceptably high bleeding risk [65,124,125].

In the general population of NSTEMI patients, loading dose with aspirin is mandatory before primary PCI, but pre-treatment with a P2Y_12_ receptor inhibitor is not routinely recommended. When used in patients with unknown coronary anatomy, the pre-treatment with a P2Y_12_ receptor inhibitor was not associated with an improvement in ischemic outcomes, but rather with a significant increase in the bleeding risk [126,127]. However, benefits were obtained when the P2Y_12_ receptor inhibitor was administered after the diagnostic coronary angiography [128], directly before PCI, showing that prasugrel and ticagrelor have a fast onset of action. If after the diagnostic coronary angiography it is found that the lesions are not amendable by PCI and the patient requires early CABG, then unnecessary bleedings are avoided by refraining from the pre-treatment with a P2Y_12_ receptor inhibitor.

Although hemophilic patients have a high bleeding risk, interventional cardiologists have rarely changed the standard ACS protocol in the presence of hemophilia. Since platelets are normal in hemophilic patients, they should receive antiplatelet agents as the general population and their administration should not be delayed in the acute setting. The international, retrospective 10-year survey of Fogarty et al. [48] showed that in 75% of cases the therapeutic protocol used for the treatment of ACS in the general population was also respected in hemophilic patients. If the replacement therapy is complete, the hemophiliacs may receive the standard regimen, including DAPT prior to PCI, with clopidogrel as the recommended P2Y_12_ receptor inhibitor due to its lower bleeding risk compared to prasugrel and ticagrelor [60].

The initial recommendations advised against the use of prasugrel and ticagrelor in hemophiliacs [64,129]. Still, there have been recently-published case reports of successful use of ticagrelor in hemophiliacs as part of DAPT (Table 1). Kacprzak et al. used ticagrelor in a difficult STEMI case requiring five stents—four EES and one BES. Ticagrelor was started with a 180 mg loading dose. Due to the high risk of stent thrombosis (all stents’ lengths were 92 mm, and narrow coronary arteries), the patient received DAPT with aspirin and ticagrelor 90 mg od for 12 months, without bleeding events under FVIII replacement therapy [119]. Vaz et al. used ticagrelor in a patient with severe hemophilia B undergoing PCI for STEMI, with a 180 mg loading dose, followed by DAPT with aspirin and ticagrelor without bleeding events [57]. After an ACS treated with PCI and stent implantation, current guidelines recommend DAPT with a potent P2Y_12_ receptor inhibitor and aspirin for a duration that varies depending on the thrombotic and hemorrhagic risk of the patient. Preventing stent thrombosis must be accomplished without causing excessive bleeding. DAPT is generally recommended for 12 months, but in patients at high risk of bleeding the duration may be reduced, but not less than 1 month [65,66]. In hemophiliacs, the ideal duration of DAPT is considered to be 1–6 months, depending on the type of stent. In an international study, DAPT with aspirin and clopidogrel was used in hemophilia patients for a duration that ranged from less than 1 month to 12 months [48,119].

It was initially considered beneficial that hemophiliacs undergoing PCI additionally receive glycoprotein IIb/IIIa (GPIIb-IIIa) inhibitors in the 12 hours after PCI [8]. However, due to the disadvantageous benefit–bleeding risk ratio observed, the use of GPIIb-IIIa inhibitors is currently allowed only in exceptional circumstances for high ischemic risk NSTEMI patients [60]. Abciximab was successfully used for the treatment of an acute thrombotic coronary occlusion during stent implantation in a patient with severe hemophilia B [58] and for bailout due to distal embolization in a patient with severe hemophilia A [53]. This indication mirrors the current guideline recommendations for the general population.

To minimize the bleeding risk during DAPT, prophylactic administration of deficient coagulation factor and of proton pump inhibitors is indicated. A strong amount of evidence to support these indications is provided by the hemophilia French registry, a case-control study that prospectively collected data on the antithrombotic treatment received by hemophilic patients with coronary artery disease and/or atrial fibrillation (AF), including 20 ACS (3 STEMI, 16 NSTEMI, and 1 unstable angina) [17]. Bleeding events were more frequent in patients on antiplatelet drugs than in controls and in patients on DAPT than on single antiplatelet therapy (SAPT). DAPT doubled the incidence of bleeding events compared to SAPT. Important, the bleedings incidence was not influenced by the antithrombotic drug used, as aspirin and clopidogrel showed similar hemorrhagic risks when used in monotherapy [17]. During antiplatelet therapy, the highest bleeding incidence was noted in patients with severe hemophilia without concomitant prophylactic factor VIII/IX administration. It was more than 16 times higher than that of patients with the same hemophilia severity, but receiving prophylaxis [17]. The significant reduction in the incidence of bleeding events in patients with severe hemophilia receiving prophylaxis highlighted the crucial role of deficient factor replacement for bleedings prevention during antiplatelet treatment in this category of patients. Still, prophylaxis did not completely counterbalance the risk of bleeding determined by antiplatelet therapy. For severe hemophilia patients on prophylaxis, bleeding risk while on antiplatelet treatment remained three-fold higher than that of controls [17]. The major beneficial effect of prophylaxis in reducing the incidence of bleeding events during antiplatelet treatment was noted in patients with moderate hemophilia as well. Therefore, regular prophylaxis should be used in both categories of patients, with severe or moderate hemophilia, to effectively protect them from major bleeding during antiplatelet treatment.

Antiplatelet agents increase the risk of bleeding regardless of the severity of hemophilia, but the more severe the hemophilia, the higher the risk. In order to lower this risk, the ADVANCE group recommended that the trough of coagulation factor levels not fall below 5–15% when the patient is on DAPT or below 1% when on aspirin alone [60]. More recent data suggests that the FVIII/FIX trough levels should be higher, ≥5%–10% during SAPT [80] and ≥20–30% [17,81] or even ≥30% during DAPT [8,63,80,82]. However, maintaining high levels is difficult in the long term, as it would require the administration of replacement products every 1–2 days [17]. In patients with mild hemophilia and residual clotting factor level of 25% or higher, routine administration of clotting factor concentrate is not needed during DAPT, which allows DAPT extension beyond 1 month if deemed necessary [61]. Thus, in establishing the duration of DAPT, the possibility of administering replacement therapy with increased frequency must be taken into account. In hemophilia patients with inhibitors on prolonged DAPT, prophylaxis with bypassing agents beyond the first month after PCI is associated with an increased risk of thrombosis and is not recommended [8,82].

Data on emicizumab use in patients with ACS is scarce. In HAVEN 3 trial (emicizumab prophylaxis in patients who have hemophilia A without inhibitors), one patient older than 65 years, with previously undiagnosed coronary artery disease, experienced STEMI [12]. The event was considered unrelated to the study drug and the patient continued treatment with emicizumab, but specific data regarding STEMI management was not published. Gundabolu et al. reported a STEMI case of a patient on emicizumab that was managed conservatively and maintained on emicizumab during hospitalization for the acute coronary event [13].

Regarding the site of bleeding while on DAPT, the French registry showed that the hemophilia patients from the control group had almost exclusively hemarthrosis and hematoma. No gastrointestinal (GI) bleedings were reported. Instead, in hemophilia patients receiving antithrombotic therapy, 10% of the bleedings were from the GI tract. All the patients that experienced GI bleedings were receiving antiplatelet treatment, and none had protection with proton-pump inhibitors [17]. GI bleedings under antiplatelet treatment are a phenomenon observed in the general population as well. In this context, it is reasonable to administer proton-pump inhibitors throughout antiplatelet therapy to all hemophilia patients to reduce the risk of GI bleeding [60,61].

When DAPT is no longer required, long-term SAPT with aspirin or clopidogrel is generally used. Routine prophylactic coagulation factor replacement therapy is not indicated. In the absence of recurrent ecchymoses or bleeding, prophylaxis is not necessary. If the opposite happens, tailored prophylaxis is initiated [129].

In patients who do not undergo PCI, clopidogrel is generally indicated [65,130], but the use of ticagrelor has also been reported. Gundabolu et al. conservatively managed a STEMI case and used DAPT with aspirin and ticagrelor for 3 months [13].

## 4. Discussion

Age-related comorbidities in hemophilia patients are a reality of the medical practice of our times. It has long been thought that hemophiliacs are protected from the development of atherosclerosis and cardiovascular disease. However, evidence accumulated over the years revealed that cardiovascular risk factors and atherosclerotic plaques are as prevalent in hemophiliacs as in the general population. Hypertension and obesity tend to become endemic in the hemophilic population [9,39,131]. The prevalence of the cardiovascular disease varies between studies, ranging from <2% to 20% depending on the age of the hemophiliacs enrolled and the severity of their disease [72]. Ischemic heart disease is found in an increasing number of hemophilic patients, especially if they are old and with cardiovascular risk factors. Thus, it is expected that the number of acute atherothrombotic coronary events rises.

In a North American cohort, ischemic heart disease was identified even in young individuals. The prevalence ranged from 0.05% in those younger than 30 years to 15.2% in those older than 60 years [36]. There are reports according to which the prevalence of coronary artery disease, stroke, and myocardial infarction are twice as high in hemophilia patients than in non-Hispanic white males [132]. Moreover, as indicated by the data collected at the end of the 2 years of follow-up in the French registry, the hemophilic patients with ischemic or thrombogenic cardiovascular disease had a risk for a new cardiovascular event at least six times higher than those without a history of cardiovascular disease [17]. In the Danish study, the case of a male with moderate hemophilia A was recorded, with two AMI (68 years, 71 years), the second one complicated with cardiac arrest [72]. It is now very clear that the well-established cardiovascular risk factors—hypertension, diabetes mellitus, dyslipidemia and smoking—appear to neutralize the potential protective effect that a factor VIII or factor IX deficiency might exert [133].

Knowledge and technique that are constantly evolving along with the experience gained in treating acute myocardial infarction in patients from the general population tend to modulate or even change the old concepts and recommendations for the hemophiliacs (Table 2). Concerns that hemophiliacs with AMI receive less aggressive treatment due to the high risk of bleeding and, therefore, have a worse cardiovascular outcome [67], should no longer exist.

PCI is the method of choice for the treatment of STEMI and high-risk NSTEMI in both the general population and hemophilic patients, and it should be performed without delay. UFH remains the preferred anticoagulant during PCI. Radial access is currently recommended as the default access site for PCI in all patients, as it is associated with lower rates of bleeding at the site of arterial puncture and vascular complications compared to the femoral access. Moreover, the need for transfusions and mortality are lower when using the radial access instead of the femoral one.

For a long time, a BMS was preferred in hemophilia patients requiring stenting, as it facilitates a shorter duration of DAPT (1 month). It was significantly less than those 6–12 months needed for a DES. Now, the need for a shorter period of DAPT is no reason for choosing a BMS. Contemporary DES exceeded first-generation DES in efficacy as they are associated with lower rates of stent thrombosis, reinfarction, target-vessel revascularization, and cardiac death. Due to their superior clinical outcomes, it is standard practice to stent an infarction-related artery with contemporary DES. Moreover, recent studies have shown that ZES and BES are superior to BMS in terms of efficacy and safety when used with DAPT for 1 month. These new data, particularly favorable to the population at high risk of bleeding, have been rapidly implemented in the treatment of patients with hemophilia, and case reports of their successful use have begun to emerge. With the old DES, the risk of bleeding while on prolonged DAPT outweighed the benefit of a reduced risk of restenosis. However, now it is possible to use the advantages of a modern DES and lower the risk of bleeding by giving 1-month DAPT as in BMS.

It is well established that DAPT with aspirin and a P2Y_12_ receptor inhibitor is required after DES implantation. DAPT for 1 month with aspirin and clopidogrel represent the standard of care for high bleeding risk patients, as clopidogrel is generally associated with less bleeding than prasugrel and ticagrelor. However, the benefits of using prasugrel or ticagrelor as part of DAPT should not be overlooked. Ticagrelor was associated with a reduced rate of myocardial infarction, stroke, or death from vascular causes [125]. Prasugrel was associated with reduced rates of ischemic events, including stent thrombosis [124]. However, a preference toward ticagrelor is observed, which is explained by differences in the safety profiles and pharmacological properties. Prasugrel increased the risk of major bleeding, including fatal bleeding [124]. Ticagrelor did not increase the rate of overall major bleeding [125]. Moreover, ticagrelor is a reversible inhibitor of the P2Y_12_ receptor that is helpful in the case of major bleeding, while prasugrel, as well as clopidogrel, irreversibly inhibit platelet function. Therefore, ticagrelor began to be successfully used in hemophiliacs undergoing primary PCI for AMI.

In order to safely perform invasive procedures and administer antithrombotic therapy, a specific level of the deficient coagulation factor is required. Maintaining the level above the threshold value usually involves replacement factor therapy, with doses and frequency of administrations depending on the severity of the disease. For PCI, all patients should receive deficient factor concentrates to ensure a safe procedure, as very high levels of deficient coagulation factor are required. A peak level exceeding 80% is necessary during PCI and 48 h after the invasive procedure [8,63,64]. For the whole DAPT duration, the recommended trough level of the deficient coagulation factor is ≥30% [8,17,63,80], as it was shown that in less completely replaced patients the bleeding risk may outweigh the potential benefit. During SAPT, the trough factor level should be ≥5–10% [80], therefore, prophylactic replacement therapy is not usually necessary in patients with mild hemophilia, where on-demand therapy is rather used.

Emicizumab, a bispecific monoclonal antibody that binds activated FIX with FX, thus replacing the function of missing activated FVIII, is a major breakthrough in the treatment of hemophilia. First, prophylaxis of bleeding with emicizumab is superior in efficacy and safety to that with FVIII [12]. Second, in the absence of homology between emicizumab and FVIII, the risk of cross-reaction with inhibitors directed against FVIII is ruled out. Since a major complication of replacement therapy using FVIII concentrates is the development of antibodies (inhibitors), emicizumab is the preferred prophylactic treatment for hemophiliacs with inhibitors. Third, it is administrated as a subcutaneous injection once a week, or every 2 or 4 weeks, and therefore it gives a significant improvement in quality of life in patients for whom prophylaxis previously required intravenous infusion of FVIII concentrates two to three times per week [134]. And fourth, the emicizumab concentrations are stable from week 5 of treatment, which implies that patients do not require regular laboratory monitoring [135].

During invasive procedures or surgery, it is recommended that treatment with emicizumab not be interrupted or the posology altered. Still, a single dose of factor VIII concentrate was administrated prior to procedure in some patients undergoing minor surgeries. Most of the high-risk invasive procedures and major surgery were performed under prophylactic coagulation factor replacement [136]. Currently, it is not known if the level of hemostatic coverage provided by prophylactic doses of emicizumab is sufficient for PCI, given that emicizumab does not fully restore hemostasis and that a peak level of coagulation factor exceeding 80% is recommended during PCI and 48 hours after the invasive procedure. Moreover, the level of protection provided by emicizumab during DAPT is also unknown. Further specific studies are necessary.

Invasive cardiac procedures in hemophiliacs with AMI are high-risk situations that require a multidisciplinary team with a high level of expertise. It is also necessary that the facility have the infrastructure and full-spectrum resources for both interventional cardiology and treating hemophilia. Immediate availability of a specialist in hemophilia and of coagulation factor concentrates/bypassing agents enables a prompt therapeutic response. The risk of bleeding secondary to antithrombotic treatment must be balanced against the risk of thrombosis secondary to hemostatic therapy.

This study has several limitations. Firstly, the available literature is heterogeneous in terms of the number of patients included and the follow-up period, which ranged from case reports to large retrospective studies. The studies reporting PCI or CABG in hemophiliacs often included both acute and elective procedures, making it difficult to extract the necessary information. Secondly, there are not enough data to make comparisons based on the type of hemophilia possible, the extent of factor deficiency, and the presence of inhibitors. The hemophilia type can be an important confounder because even though their clinical manifestations are similar, it is generally considered that bleeding episodes in patients with hemophilia A are more severe and occur at a higher frequency than in patients with hemophilia B. Moreover, patients with inhibitors receive bypassing agents and not coagulation factor concentrates, therefore, they may be more prone to thrombotic events. Thirdly, hemophiliacs have been excluded from randomized clinical trials, therefore, the standard management approach is based on institutional protocols and consensus of experts.

## 5. Conclusions

The management of acute myocardial infarction in hemophilic patients requires a team effort. The collaboration between an experienced interventional cardiologist and an expert in hemophilia is mandatory, as the fragile balance between thrombosis and bleeding must be kept under tight surveillance and control. There is a tendency to follow the recommendations of the guidelines for the general population in hemophiliacs in terms of time-frame, procedure, devices, and drug therapy. It is the general opinion that the hemophilic patient with AMI should benefit from the technological and therapeutic progress, under the protection given by an adequate replacement of the deficient coagulation factor and intensive monitoring. A guideline that harmonizes local protocols and includes the latest available data is highly necessary and expected.

## Figures and Tables

**Table 1:** Use of new-generation stents and potent P2Y_12_ receptor inhibitors in hemophilia patients with acute myocardial infarction.

Author, Year	Sex, Age	Hemophilia Type and Severity	Comorbidities	Type of Acute Myocardial Infarction	Case Management
Theodoropoulos et al. 2021 [55]	Male, 70 years	B, mild	Hypertension, COPD	NSTEMI	Aspirin and clopidogrel—loading doses PCI—radial access Zotarolimus eluting stent UFH periprocedural DAPT 1 month (aspirin and clopidogrel) Prophylactic administration of FIX: for PCI and during DAPT
Vaz et al. 2021 [57]	Male, 56 years	B, severe	Hypertension, Dyslipidemia, Smoking	STEMI	Aspirin—loading dose PCI—radial access Ticagrelor 180 mg and UFH 5.000 UI iv. at the time of PCI DES was implanted DAPT with aspirin and ticagrelor, then SAPT with aspirin Symptoms occured 8 h after FIX concentrate infusion; Prophylactic administration of FIX: during DAPT
Gundabolu et al. 2019 [13]	Male, 40 years	A, severe with inhibitors	Smoking	STEMI	Medical management Low-dose UFH: 5–10 U/kg/h (without a bolus), for 4 days DAPT with aspirin and ticagrelor for 3 months, then SAPT with aspirin Replacement treatment administrated before STEMI occurrence: Emicizumab 1.5 mg/kg 5 days before rVIIa 100 mcg/kg 36 h before. During hospitalization, emicizumab was continued, but rVIIa was not used.
Kacprzak et al. 2018 [119]	Male, 67 years	A, severe	Chronic hepatitis C	STEMI	Aspirin and clopidogrel—loading doses 5000 IU UFH iv PCI—radial access 5 DES: 4 everolimus eluting stents and 1 biolimus A9 eluting stent After stenting: clopidogrel was switched to ticagrelor (180 mg loading dose) DAPT with aspirin and ticagrelor for 12 months, then SAPT with aspirin FVIII prophylaxis was given during hospitalization 30 months of follow-up: no bleeding episodes.
Bailly et al. 2018 [51]	Male, 54 years	A, severe	Dyslipidemia, Smoking HIV infection	STEMI	PCI Zotarolimus stent DAPT with aspirin and clopidogrel >12 months FVIII prophylaxis before PCI

COPD = chronic obstructive pulmonary disease; STEMI = ST-elevation myocardial infarction; NSTEMI = non-ST-elevation myocardial infarction; PCI = percutaneous coronary intervention; UFH = unfractionated heparin; DAPT = dual antiplatelet therapy; SAPT = single antiplatelet therapy; DES = drug-eluting stent; rVIIa = recombinant factor VII activate; FVIII = coagulation factor VIII; FIX = coagulation factor IX; HIV = human immunodeficiency virus.

**Table 2:** The main recommendations for the treatment of AMI in patients with hemophilia.

	Recommendation	References
Reperfusion therapy	PCI is the main line of treatment for STEMI and high-risk NSTEMI hemophiliacs, and it should be performed as soon as possible.	[8,61,62]
	CABG is reserved for patients with complex coronary artery disease as a three-vessel disease, left main stenosis or stenosis of the proximal left anterior descending artery.	[48,60,63,69]
Anticoagulant	UFH is the anticoagulant of choice during PCI.	[60,65]
Access site for PCI	Radial access is the default access site for PCI. Femoral access may be used if the interventional cardiologist does not have experience in radial access.	[60,61]
Stent	New-generation DES requiring short DAPT may be used (zotarolimus-eluting stent, everolimus-eluting stent, biolimus A9-eluting stent).	[51,55,118, 119]
	Hemophiliacs with mild disease—factor levels exceeding 25%—could receive DES as they may not require administration of coagulation factor concentrates for the duration of DAPT.	[61]
Antiplatelet therapy	Dual antiplatelet therapy should be administered for as short a duration as possible. The ideal duration of DAPT is considered to be 1–6 months, depending on the type of stent.	[48,60,65, 66,119]
	DAPT for 1 month is recommended with new-generation DES (zotarolimus-eluting stent, biolimus A9-eluting stent).	[55,120,121,122]
	Clopidogrel is the preferred P2Y_12_ receptor inhibitor as part of DAPT.	[60]
	Ticagrelor may be used as part of DAPT.	[57,119]
	Aspirin or clopidogrel may be used for SAPT.	[13,57,119]
Replacement coagulation factor therapy	Replacement coagulation factor therapy should aim for a peak level of at least 80% during PCI and for 48 h after PCI.	[8,17,62,63, 80,81,82]
	FVIII/FIX trough levels should be ≥30% during DAPT.	[8,63,80,82]
	FVIII/FIX trough levels should be ≥5%–10% during SAPT.	[80]
PPI	All hemophilia patients should receive proton-pump inhibitors throughout DAPT to reduce the risk of GI bleeding.	[60,61]

PCI = percutaneous coronary intervention; STEMI = ST-elevation myocardial infarction; NSTEMI = non ST-elevation myocardial infarction; CABG = coronary artery bypass grafting; UFH = unfractionated heparin; DES = drug-eluting stent; DAPT = dual antiplatelet therapy; SAPT = single antiplatelet therapy; FVIII = coagulation factor VIII; FIX = coagulation factor IX; GI = gastrointestinal.
